# Mutational Analysis of *ATP8B1* in Patients with Chronic Pancreatitis

**DOI:** 10.1371/journal.pone.0080553

**Published:** 2013-11-19

**Authors:** Wendy L. van der Woerd, Désirée Y. van Haaften-Visser, Stan F. J. van de Graaf, Claude Férec, Emmanuelle Masson, Janneke M. Stapelbroek, Peter Bugert, Heiko Witt, Roderick H. J. Houwen

**Affiliations:** 1 Department of Pediatric Gastroenterology, Wilhelmina Children's Hospital, University Medical Center Utrecht, Utrecht, The Netherlands; 2 Department of Metabolic Diseases, University Medical Center Utrecht, Utrecht, The Netherlands; 3 Laboratoire de Génétique Moléculaire et d'Histocompatibilité, Centre Hospitalier Régional Universitaire (CHRU) Brest, Brest, France; 4 Institut National de la Santé et de la Recherche Médicale (INSERM), U1078, Brest, France; 5 Institute of Transfusion Medicine and Immunology, Medical Faculty Mannheim, Heidelberg University, German Red Cross Blood Service of Baden-Württemberg-Hessen, Mannheim, Germany; 6 Department of Pediatrics, Technische Universität München (TUM), Munich, Germany; 7 Else Kröner-Fresenius-Zentrum für Ernährungsmedizin (EKFZ) & Zentralinstitut für Ernährungs- und Lebensmittelforschung (ZIEL), Technische Universität München (TUM), Freising, Germany; IFOM, Fondazione Istituto FIRC di Oncologia Molecolare, Italy

## Abstract

**Background:**

Mutations in genes encoding cationic trypsinogen (*PRSS1*), pancreatic secretory trypsin inhibitor (*SPINK1*) and chymotrypsinogen C (*CTRC*) are associated with chronic pancreatitis. However, in many patients with a familial chronic pancreatitis pattern suggesting a genetic cause, no mutations in either of these genes can be found, indicating that other, still unknown, associated genes exist. In this respect *ATP8B1* is an interesting candidate due to its strong expression in the pancreas, its supposed general function in membrane organization and the higher incidence of pancreatitis in patients with ATP8B1 deficiency.

**Methods:**

We analyzed all 27 *ATP8B1* coding exons and adjacent non-coding sequences of 507 chronic pancreatitis patients by direct sequencing. Exons that harbored possible relevant variations were subsequently sequenced in 1,027 healthy controls.

**Results:**

In the exonic regions, 5 novel non-synonymous alterations were detected as well as 14 previously described alterations of which some were associated with ATP8B1 deficiency. However, allele frequencies for any of these variations did not significantly differ between patients and controls. Furthermore, several non-synonymous variants were exclusively detected in control subjects and multiple variants in the non-coding sequence were identified with similar frequencies in both groups.

**Conclusions:**

We did not find an association between heterozygous *ATP8B1* variants and chronic pancreatitis in our cohort of patients with hereditary and idiopathic chronic pancreatitis.

## Introduction

Chronic pancreatitis (CP) is an inflammatory disease characterized by destruction of pancreatic parenchyma that can result in permanent impairment of both exocrine and endocrine pancreatic function [1]. CP might cluster in families, and in many of these affected subjects as well as young patients without a family history of pancreatitis, mutations in the genes coding for cationic trypsinogen (*PRSS1*), pancreatic secretory trypsin inhibitor (*SPINK1*) and chymotrypsinogen C (*CTRC*) can be identified [2]–[5]. In general, mutations in these genes disturb the protease-antiprotease equilibrium within the pancreas, either through enhanced activation of trypsinogen or a reduced inhibition of this activated protease. *CFTR* mutations too enhance the susceptibility for idiopathic chronic pancreatitis [6]. Despite the growing number of genes associated with CP, in many patients with pancreatitis and an inheritance pattern suggesting a genetic cause, no variant within these genes can be identified, suggesting that other still unidentified genes might exist [1].

ATP8B1 deficiency is an autosomal recessive disease characterized by mutations in *ATP8B1* (formerly designated as *FIC1*) [7]. ATP8B1 deficiency can present with persistent cholestasis, usually at young age (progressive familial intrahepatic cholestasis; PFIC) or with episodic cholestasis at any age (benign recurrent intrahepatic cholestasis; BRIC). Occasionally the benign variant will progress to the more severe and permanent form of intrahepatic cholestasis, indicative of a clinical continuum [8]. Extrahepatic manifestations such as diarrhoea, pancreatitis and hearing loss can be observed in patients with ATP8B1 deficiency [9]. ATP8B1 is thought to be essential for maintaining membrane lipid asymmetry by translocation of aminophospholipids from the outer to the inner leaflet of the plasmamembrane. Loss of this asymmetric distribution of phospholipids in cellular membranes is presumed to affect fundamental processes such as membrane transport. Therefore deficiency of ATP8B1 might result in dysfunction of transmembrane transporters such as ABCB11, the bile salt export pump, within the canalicular membrane of liver cells, causing intrahepatic cholestasis [8]. Similarly, the stability of the cellular membranes in cochlear hair cells is reduced in patients with ATP8B1 deficiency, resulting in progressive hearing loss [10]. Apart from liver and cochlear cells, ATP8B1 is also expressed in other tissues, especially the pancreas [7], [10], [11]. As the incidence of pancreatitis is higher in patients with ATP8B1 deficiency, we hypothesized that mutations in this gene might also be associated with CP [9], [12], [13]. Therefore, we investigated a large cohort of CP patients and control subjects for *ATP8B1* mutations.

## Materials and Methods

### Study subjects

The study was approved by the local ethics committee of the Technische Universität München and the ethical review committee of the Université de Bretagne Occidentale. All study subjects gave their written informed consent for genetic analysis. For this study, 507 patients with hereditary or idiopathic chronic pancreatitis were included. The patients originated from Germany (*n* = 316) and France (*n* = 191). In the German patients, the diagnosis of CP was based on two or more of the following findings as described previously: presence of a typical history of recurrent pancreatitis, pancreatic calcifications and/or pancreatic ductal irregularities revealed by endoscopic retrograde pancreaticography or by magnetic resonance imaging of the pancreas, and pathological sonographic findings [5]. Hereditary pancreatitis was diagnosed when one first-degree relative or two or more second-degree relatives suffered from recurrent acute or chronic pancreatitis without apparent precipitating factors. Affected individuals were classified as having idiopathic chronic pancreatitis in the presence of such a family history and when known precipitating factors, such as alcohol abuse, trauma, medication, infection, metabolic disorders, were absent.

In the French patients, CP was diagnosed as described previously [3],[14]. Patients with hereditary pancreatitis had three or more affected family members involving at least two generations.

The control group consisted of 1,027 unrelated healthy individuals of German (*n* = 742) and French (*n* = 285) origin. Whenever a non-synonymous variation in the coding sequence was encountered all 1,027 controls were sequenced for the exon involved.

### Mutational analysis

Genomic DNA of peripheral blood leukocytes was extracted routinely. Primer pairs for PCR were designed to amplify all 27 coding exons, with flanking intron-exon boundaries of *ATP8B1* followed by uni-directional DNA sequencing. Primer sequences are available on request. PCR was performed using standard methodology and semi-automated sequence analysis using an ABI 3730 sequencer (Applied Biosystems).

To detect nucleotide sequence changes of potential relevance to clinical phenotypes, we analyzed the sequence output of the patient cohort for variants that resulted in amino acid changes, nonsense variants or deletions/insertions. Exons with one of these alterations were subsequently analyzed in the control population by DNA sequencing.

The reference sequence was derived from GenBank (http://www.ncbi.nlm.nih.gov/entrez, reference sequence NM_005603.4). The A of the ATG start codon was used as nucleotide +1. The mutations are described according to the nomenclature recommended by the Human Genome Variation Society (http://www.hgvs.org/mutnomen).

### Statistical analysis

The significance of the differences between mutation frequencies between patients and controls was tested by two-tailed Fisher's exact test. A p value of less than 0.05 was considered significant. Correction according to Bonferroni was carried out.

## Results

In our cohort of 507 CP patients we identified 19 different *ATP8B1* variants leading to an amino acid change (Table 1). Five (p.R833W, p.K885T, p.R946T, p.1150T, p.V1197L) were not described before, neither in the published literature nor in the Human Genome Mutation Database. Fourteen variants were described earlier; three of these variants (p.Y500H, p.E665X, p.A1208fs) are associated with the progressive form of ATP8B1 deficiency and six with the episodic form (p.N45T, p.H78Q, p.D70N, p.E429A, p.I577V, p.M674T). Four were mentioned in the literature for their possible association with intrahepatic cholestasis of pregnancy (p.N45T, p.D70N, p.K203E, p.F305I). In two patients more than one *ATP8B1* variant was detected. The first patient had three non-synonymous variants (p.H78Q, p.I577V, p.M674T), all possibly associated in literature with episodic ATP8B1 deficiency. The second patient had two variants (p.E665X, p.A1208fs), both described in patients with the progressive form of ATP8B1 deficiency. Interestingly both these patients presented with chronic pancreatitis and had no signs of liver disease whatsoever.

**Table 1:** Non-synonymous exonic *ATP8B1* variations in CP patients and controls.

Region	Nucleotide Change	Amino Acid Change	Genotype	Patients (%)	Controls (%)	P value
Exon 2	c.134A>C	p.N45T	CC	1/507 (0.2)	0/1027 (0)	0.33
			AC	7/507 (1.4)	17/1027 (1.7)	0.83
Exon 3	c.208G>A	p.D70N	GA	8/507 (1.6)	9/1027 (0.9)	0.3
	c.234C>G	p.H78Q	CG	1/507 (0.2)	0/1027 (0)	0.33
Exon 7	c.607A>G	p.K203E	AG	2/507 (0.4)	3/1027 (0.3)	0.67
Exon 10	c.913T>A	p.F305I	TA	1/507 (0.2)	8/1027 (0.8)	0.29
Exon 12	c.1046T>C	p.I349T	TC	0/507 (0)	1/1027 (0.1)	0.33
	c.1102A>G	p.N368D	AG	0/507 (0)	1/1027 (0.1)	0.33
	c.1177A>G	p.I393V	AG	5/507(1.0)	6/1027 (0.6)	0.52
Exon 13	c.1286A>C	p.E429A	AC	1/507 (0.2)	0/1027 (0)	0.33
	c.1405T>G	p.C469G	TG	0/507 (0)	1/1027 (0.1)	0.33
Exon 15	c.1498T>C	p.Y500H	TC	1/507 (0.2)	3/1027 (0.3)	1.0
	c.1603C>A	p.H535N	CA	0/507 (0)	1/1027 (0.1)	0.33
Exon 16	c.1729A>G	p.I577V	AG	1/507 (0.2)	0/1027 (0)	0.33
Exon 18	c.1982T>C	p.I661T	TC	0/507 (0)	2/1027 (0.2)	0.55
	c.1993G>T	p.E665X	GT	1/507 (0.2)	0/1027 (0)	0.33
	c.2021T>C	p.M674T	TC	1/507 (0.2)	0/1027 (0)	0.33
Exon 22	c.2442G>T	p.K814N	GT	0/507 (0)	1/1027 (0.1)	0.33
	c.2497C>T	p.R833W	CT	1/507 (0.2)	0/1027 (0)	0.33
	c.2654A>C	p.K885T	AC	1/507 (0.2)	0/1027 (0)	0.33
Exon 23	c.2771A>G	p.Y924C	AG	0/507 (0)	1/1027 (0.1)	0.33
	c.2789G>A	p.R930Q	GA	1/507 (0.2)	0/1027 (0)	0.33
	c.2837G>C	p.R946T	GC	1/507 (0.2)	0/1027 (0)	0.33
	c.2855G>A	p.R952Q	AA	7/507 (1.4)	13/1027 (1.3)	0.82
			GA	95/507 (18.7)	196/1027 (19.1)	0.89
	c.2927C>A	p.A976E	CA	0/507 (0)	1/1027 (0.1)	0.33
Exon 27	c.3449T>C	p.I1150T	TC	1/507 (0.2)	0/1027 (0)	0.33
Exon 28	c.3589G>T	p.V1197L	GT	1/507 (0.2)	0/1027 (0)	0.33
	c.3622_3628delGCCTACG	p.A1208fs		1/507(0.2)	0/1027 (0)	0.33
All exons	-	-	-	139/507 (27.4)	264/1027 (25.7)	0.5

CP = chronic pancreatitis.

Those exons in which a non-synonymous variant was detected in CP patients (13 exons) were also sequenced in our control cohort of 1,027 subjects. No alteration was significantly overrepresented in the patient group. Also the combined frequency of these non-synonymous exonic *ATP8B1* variations in CP patients did not significantly differ from that in controls (p = 0.5). Furthermore, in the control population we detected 8 additional non-synonymous variations, of which 5 had not been described before (Table 1). In addition, synonymous or non-coding sequence variations were detected in both groups (Table 2 and 3). There was no significant difference for any of these variants between the CP patients and control group except for the SNP c.2097+89T>C. However, after using the Bonferroni correction for multiple testing, this significance disappeared.

**Table 2:** Synonymous exonic *ATP8B1* variations in CP patients and controls.

Region	Nucleotide Change	Amino Acid Change	Genotype	Patients (%)	Controls (%)	P value
Exon 3	c.189A>C	p.T63T	AC	1/507 (0.2)	0/1027 (0)	0.33
	c.246A>G	p.T82T	AG	8/507 (1.6)	14/1027 (1.4)	0.82
	c.276T>C	p.Y92Y	TC	0/507 (0)	1/1027 (0.1)	0.33
Exon 10	c.811A>C	p.R271R	CC	505/507 (99.6)	1027/1027 (100)	0.11
			AC	2/507 (0.4)	0/1027 (0)	0.11
Exon 12	c.1101C>A	p.G367G	CA	0/507 (0)	1/1027 (0.1)	0.33
Exon 14	c.1461C>T	p.H478H	CT	1/507 (0.2)	NS	-
Exon 16	c.1710C>T	p.L570L	CT	1/507 (0.2)	0/1027 (0)	0.33
Exon 18	c.2052C>T	p.D684D	CT	0/507 (0)	1/1027 (0.1)	0.33
Exon 22	c.2664G>A	p.T888T	GA	0/507 (0)	1/1027 (0.1)	0.33
Exon 23	c.2763G>A	p.S921S	GA	0/507 (0)	1/1027 (0.1)	0.33
	c.2880C>T	p.A960A	CT	0/507 (0)	1/1027 (0.1)	0.33
Exon 28	c.3669C>T	p.S1223S	CT	1/507 (0.2)	0/1027 (0)	0.33
	c.3699G>A	p.P1233P	GA	1/507 (0.2)	0/1027 (0)	0.33

CP = chronic pancreatitis; NS = no sequence data available.

**Table 3:** *ATP8B1* variations in non-coding regions in CP patients and controls.

Region	Nucleotide Change	Genotype	Patients (%)	Controls (%)	P value
*Promotor variation*					
Promotor	c.-4C>G	CG	0/507 (0)	1/1027 (0.1)	0.33
*Intronic variations*					
Intron 2	c.182-5T>A	TA	1/507 (0.2)	0/1027 (0)	0.33
	c.182-72G>A	GA	80/507 (15.8)	184/1027 (17.9)	0.31
		AA	3/507 (0.6)	19/1027 (1.9)	0.07
Intron 6	c.555-3T>C	TC	0/507 (0)	1/1027 (0.1)	0.33
Intron 7	c.628-30G>A	GA	0/507 (0)	2/564 (0.4)	0.5
	c.628-31C>T	CT	1/507 (0.2)	0/564 (0)	0.47
Intron 8	c.698+20C>T	CT	251/507 (49.5)	263/564 (46.6)	0.36
		TT	118/507 (23.3)	110/564 (19.5)	0.14
Intron 9	c.782-34G>A	GA	1/507 (0.2)	0/1027 (0)	0.33
Intron 12	c.1221-8C>G	CG	1/507 (0.2)	0/1027 (0)	0.33
Intron 13	c.1429+49G>A	GA	1/507 (0.2)	2/1027 (0.2)	1.0
	c.1430-42A>G	AG	135/507 (26.6)	NS	-
		GG	17/507 (3.4)	NS	-
Intron15	c.1631-10T>A	TA	0/507 (0)	1/1027 (0.1)	0.33
	c.1637-37T>C	TC	2/507 (0.4)	6/1027 (0.6)	1.0
Intron 16	c.1820-27G>A	GA	1/507 (0.2)	NS	-
Intron 18	c.2097+60T>G	TG	0/507 (0)	3/1027 (0.3)	0.56
	c.2097+89T>C	TC	30/507 (5.9)	32/1027 (3.1)	0.01
		CC	3/507 (0.6)	0/1027 (0)	0.04
	c.2097+97T>G	TG	0/507 (0)	1/1027 (0.1)	0.33
Intron 20	c.2285+29C>T	CT	206/507 (40.6)	NS	-
		TT	31/507 (6.1)	NS	-
	c.2285+32A>G	AG	3/507 (0.6)	NS	-
Intron 22	c.2707+9T>G	TG	0/507 (0)	1/1027 (0.1)	0.33
	c.2707+43A>G	AG	0/507 (0)	1/1027 (0.1)	0.33
	c.2709-59T>C	TC	0/507 (0)	2/1027 (0.2)	0.55
Intron 23	c.2931+9A>G	AG	0/507 (0)	1/1027 (0.1)	0.33
	c.2931+59T>A	TA	179/507 (35.3)	412/1027 (40.1)	0.07
		AA	43/507 (8.5)	90/1027 (8.8)	0.92
Intron 24	c.3016-9C>A	CA	3/507 (0.6)	NS	-
Intron 27	c.3531+8G>T	GT	137/507 (27.0)	282/1027 (27.5)	0.9
		TT	17/507 (3.4)	33/1027 (3.2)	0.88
	c.3532-15C>T	CT	186/507 (36.7)	422/1027 (41.1)	0.11
		TT	41/507 (8.1)	84/1027 (8.2)	1.0
*3′flanking region (3′FR)*					
3′FR	c.*11C>T	CT	13/507 (2.6)	25/1027 (2.4)	0.86

CP = chronic pancreatitis; NS = no sequence data available.

## Discussion

At the outset of our investigations, *ATP8B1* seemed a plausible candidate gene for chronic pancreatitis due to its high expression in the pancreas, its supposed general function in membrane organization and the finding that 2 out of 10 individuals affected with ATP8B1 deficiency had chronic pancreatitis [12]. However, we did not find an association between heterozygous *ATP8B1* variants and hereditary or idiopathic chronic pancreatitis when comparing 507 patients and 1,027 controls.

We did identify two CP patients with two or three non-synonymous *ATP8B1* variants. We could not experimentally verify independent inheritance as no genomic material was available from parents or unaffected family members. However if these patients were indeed compound heterozygous, these genotypes are predicted to result in an ATP8B1 deficiency phenotype. Especially the p.E665X and p.A1208fs mutations change the structure of ATP8B1 significantly and can cause PFIC. Yet these two CP patients did not have any signs of liver disease or extrahepatic features of ATP8B1 deficiency other than pancreatitis. ATP8B1 deficiency without liver disease has been described before, suggesting that reduced penetrance of the liver phenotype can indeed be seen [15]. Other factors as modifier genes and environmental factors may also contribute to this phenomenon. Our findings are compatible with a model in which CP can be caused by mutations in ATP8B1 on both alleles, which is in line with the frequent occurrence of pancreatitis in patients with ATP8B1 deficiency. Pancreatitis might even be the only symptom in patients with ATP8B1 deficiency.

In addition, our data do contribute to a better understanding of the role of rare heterozygous *ATP8B1* variants in health and disease. For example p.D70N was previously suggested to contribute to the etiology of intrahepatic cholestasis of pregnancy (ICP) as 3/182 ICP patients harbored this variant and none of 120 controls [16]. Similarly p.N45T and p.K203E were each found in one ICP patient and in none of 100 controls [17]. Our current data, giving a frequency of respectively 0.9% for p.D70N, 1.7% for p.N45T and 0.3% for p.K203E in a cohort of over 1,000 healthy controls, suggest that these earlier findings might very well have been caused by statistical variation in a relatively small control cohort.

In conclusion, our investigation did not reveal an association between heterozygous *ATP8B1* variants and hereditary or idiopathic chronic pancreatitis. However it suggests that pancreatitis might be the first or sole symptom of ATP8B1 deficiency. Furthermore earlier suggestions of an involvement of ATP8B1 variants in ICP might have been due to a chance effect.
